# Fibronectin Glomerulopathy Without Typical Renal Biopsy Features in a 4-Year-Old Girl with Incidentally Discovered Proteinuria and a G417V *FN1* Gene Mutation

**DOI:** 10.3390/ijms26020641

**Published:** 2025-01-14

**Authors:** Tibor Kalmár, Dániel Jakab, Zoltán Maróti, Gyula Pásztor, Sándor Turkevi-Nagy, Éva Kemény, Helmut Hopfer, Jan Ulrich Becker, Csaba Bereczki, Béla Iványi

**Affiliations:** 1 Genetic Diagnostic Laboratory, Department of Pediatrics, Albert Szent-Györgyi Medical Center, Faculty of Medicine, University of Szeged, 6720 Szeged, Hungary; 2 Department of Pediatrics, Albert Szent-Györgyi Medical Center, Faculty of Medicine, University of Szeged, 6720 Szeged, Hungary; 3 Department of Radiology, Albert Szent-Györgyi Medical Center, Faculty of Medicine, University of Szeged, 6720 Szeged, Hungary; 4 Department of Pathology, Albert Szent-Györgyi Medical Center, Faculty of Medicine, University of Szeged, 6720 Szeged, Hungary; turkevi-nagy.sandor@med.u-szeged.hu (S.T.-N.); ivanyi.bela@med.u-szeged.hu (B.I.); 5 Institut für Pathologie, Unversitätsspital Basel, 4031 Basel, Switzerland; 6 Institut für Allgemeine Pathologie und Pathologische Anatomie, Uniklinik Köln, 50937 Köln, Germany

**Keywords:** children, chronic kidney disease, clinical genetics, fibronectin glomerulopathy, fibronectin 1 gene, glomerular disease, Ki-67, mesangial cell proliferation, proteinuria, whole exome sequencing

## Abstract

Fibronectin glomerulopathy (FG) is caused by fibronectin 1 (*FN1*) gene mutations. A renal biopsy was performed on a 4-year-old girl with incidentally discovered proteinuria (150 mg/dL); her family history of renal disease was negative. Markedly enlarged glomeruli (mean glomerular diameter: 196 μm; age-matched controls: 140 μm), α-SMA-positive and Ki-67-positive mesangial cell proliferation (glomerular proliferation index 1.76), the mild expansion of mesangial areas, no immune or electron-dense deposits, normal glomerular basement membrane, and diffusely effaced foot processes were observed. Genetic testing identified a de novo heterozygous mutation (Gly417Val) in the collagen-binding site of the FN II-2 domain, prompting fibronectin immunostaining. Strong mesangial positivity was noted, hence FG was diagnosed. The follow-up period of 29 months revealed nephrotic range proteinuria, intermittent microhematuria, glomerular hyperfiltration, and preserved renal function. The biopsy features of early childhood-onset FG were compared to a case of FG with a lobular pattern diagnosed in a 44-year-old patient with undulating proteinuria, microhematuria, hypertension known for a year, and a positive family history. Early childhood-onset FG was characterized by glomerular enlargement, mesangial proliferation, and no changes that suggested fibronectin deposition disease. In summary, the novel aspects of the case were that the mutation was located at the collagen-binding site of the *FN1* gene, not identified earlier, and the histologic spectrum of FG was expanded by the observed mesangial proliferative pattern and striking glomerulomegaly. Now, FG should also be considered among the monogenic causes of proteinuric kidney diseases in pediatric nephrology practice.

## 1. Introduction

Fibronectin (FN) is a large dimeric extracellular matrix (ECM) glycoprotein consisting of two monomers linked by disulfide bonds. Each monomer contains three subunits termed FN I, FN II, and FN III domains. FN functions as an adhesive molecule and binds matrix metalloproteinases, growth factors, collagen I, cell surface integrin receptors, heparin, fibulin, fibrin, and so on. It also plays a role in cell adhesion, migration, and the control of the cell cytoskeleton and differentiation. FN exists in two isoforms. The plasma form is produced by the hepatocytes, it circulates in the blood, and with tissue injury, it is incorporated into fibrin clots. The cellular form is secreted by various cells and assembled into insoluble fibrils in extracellular matrices and basement membranes [1,2,3]. Histologic studies on the location of FN in the human kidney demonstrated strong staining in the mesangium and lighter staining in the Bowman’s capsule and tubular basement membrane. With electron microscopy (EM), the junction between the foot processes of podocytes and the glomerular basement membrane (GBM) also exhibited positivity [4].

Fibronectin glomerulopathy (FG) is a rare autosomal dominant kidney disease with age-related penetrance [5,6]. FG is commonly associated with pathogenic mutations in the FN-encoding *FN1* gene [6]. However, not all cases of FG have an *FN1* gene abnormality [7,8]. Pathogenic variants of *FN1* are mostly located in the heparin-binding domains that play a role in FN-cell interactions and FN fibrillogenesis [6,9,10,11,12,13,14,15,16,17,18,19]. Pathogenic variants in the integrin binding domain are infrequent [10,20,21]. Up until now, the collagen-binding site of the FN II-2 domain has not been described as a site of pathogenic mutation. Although more than two-thirds of FG patients have a positive family history [7], not all family members with the mutation develop clinical kidney disease [10,22], suggesting that an additional hypomorphic mutation seems to be necessary for the clinical disease [23]. FG manifests itself usually in the second and third decade of life with varying degrees of proteinuria, often within the nephrotic range, with or without microhematuria and hypertension. The age of the youngest and the oldest patient diagnosed with FG so far is 2 years and 88 years, respectively [24,25]. The serum FN levels are normal [5,26,27,28]. FG is generally slowly progressive, and it reaches end-stage renal failure within 15–20 years of the onset of the disease [29]. There is no specific therapy for FG so far. Control of blood pressure and proteinuria with angiotensin-converting enzyme (ACE) inhibitors or angiotensin II receptor blockers are the cornerstones of nonspecific treatment methods. The reduction in proteinuria was observed in a patient with FG after a 6-month course of treatment with prednisone [18]. FG may recur in kidney transplants [16,22,30,31].

Light microscopically, FG manifests itself either by the lobular pattern [5,32,33,34,35] or the membranoproliferative glomerulonephritis (MPGN)-like pattern [16,35]. More common is the lobular pattern. Diffuse and severe mesangial and variable subendothelial deposits are seen, accompanied by minimal to mild mesangial hypercellularity. The deposits are periodic acid-Schiff (PAS)-positive, silver methenamine-negative, and stained red with trichrome. With EM, the deposited material is markedly electron-dense and fine granular, and it may contain fibrils between 12 and 16 nm in diameter [27]. The real clue for the diagnosis is the strong immunohistochemical positivity of deposits with antisera to FN [5,26,27,28].

The pathogenesis of FG is poorly understood. It is not known how changes in *FN1* lead to the deposition of FN in glomeruli [36]. A mass spectrometry-based analysis of dissected glomeruli in FG revealed that the deposits contained large amounts of ECM protein fibulin-1 [22,30] or fibulin-1 and fibulin-5 [37]. The fibulins bind to and regulate the functions of FN. It is hypothesized that plasma-derived mutant FN protein–fibulin complexes accumulate progressively in the mesangial matrix because the clearance of these complexes is probably defective. The deposited complexes impair normal, FN-dependent glomerular intrinsic cell motility, adhesion, and spreading and they exert an effect on endothelial cell spreading and podocyte cytoskeleton reorganization [6,23,28,36].

Here, we present the case of a 4-year-old girl evaluated with renal biopsy intervention for asymptomatic proteinuria. The findings were insufficient for a definite diagnosis. Genetic testing explored a likely pathogenetic variant of the *FN1* gene and prompted immunostaining for FN, and the result of the latter concluded the case as FG. The biopsy features of this pediatric case were then compared with that of typical FG in an adult patient diagnosed in our institute earlier. A comparison, along with the survey of the literature data, delineated a hitherto not-described histopathological pattern of FG, i.e., the mesangial proliferative pattern. Reports on the features of early childhood-onset FG are rare [16,24,38], hence the clinical, morphological, and genetic features of our case might be of interest to nephrologists, nephropathologists, and geneticists.

## 2. Case Presentation

### 2.1. Medical History, Clinical Findings, and Follow-Up

The evaluation of a 2.5-year-old Caucasian girl in a local hospital revealed hypertension without any obvious secondary cause, and ACE inhibitor enalapril treatment was started (the reason for the medical check-up could not be retrieved from the records; there were no data for urinalysis). At the age of four, mild fever and no other complaints were noted by her mother, a nurse by profession, who performed a urine sulfosalicylic acid test that detected proteinuria. The girl was then referred to our clinic for further evaluation. On admission, the physical examination was unremarkable. The laboratory assessment revealed serum ions in the normal range, a decreased albumin value, an increased eGFR value, a cholesterol value of 10.44 mmol/L, and a triglyceride value of 1.8 mmol/L. The serological tests for hepatitis virus A, B, and C infection and antinuclear antibodies, anti-double-stranded DNA antibodies, and anti-neutrophilic cytoplasmic antibodies proved negative. The levels of complement factors C3 and C4 were in the normal range. The urine test revealed non-nephrotic range proteinuria. Hypercalciuria was checked, with a negative result. The trans-tubular phosphate reabsorption was retained (97.6%). The urinalysis proved negative. The abdominal ultrasonography described normal-sized kidneys with normal echogenicity. The family history of bilateral chronic kidney disease was also negative. The patient was ordered back for close follow-up visits. Since the amount of proteinuria displayed an increasing tendency, she was admitted for a kidney biopsy evaluation. The histopathological findings did not match any diagnostic entity. The genetic testing identified a likely pathogenic variant in the *FN1* gene, and the immunostaining for glomerular FN deposition concluded the case as FG. She was treated with enalapril, beta-blocker bisoprolol, and vitamin D intake.

During the follow-up period of 29 months, the proteinuria rose to the nephrotic range despite enalapril treatment, and intermittent microhematuria were recorded. Generalized edema was not detected, and she remained normotensive. The serum albumin levels were moderately decreased. The elevated serum triglyceride and cholesterol levels were attributed to hypoalbuminemia. The eGFR values were consistently and conspicuously elevated. The laboratory results of the kidney disease are summarized in Table 1. Currently, the introduction of dapagliflozin is meant to attenuate the rate of glomerular hyperfiltration and the amount of proteinuria (this therapeutic modality is under license by the Hungarian medical authorities).

### 2.2. Renal Biopsy Evaluation

The biopsy specimen was processed for light microscopy (LM), immunofluorescence (IF), and EM. For LM, the formalin-fixed and paraffin-embedded (FFPE) tissue samples were stained with hematoxylin and eosin (HE), periodic acid-Schiff (PAS), Masson’s trichrome, acid fuchsin orange G, methenamine silver of Jones, and Congo red. In addition, the frozen sections prepared for IF were stained with HE, PAS, Masson’s trichrome, and Oil Red O. For IF, the frozen sections were incubated with FITC-conjugated antibodies to IgG, IgA, IgM, kappa, lambda, complement (C) 3, C1q, and fibrinogen (Dako, Denmark). Three immunostainings were performed on FFPE sections. Mesangial cell activation and proliferation [39] were assessed in stains for α-SMA (1:300; clone 1A4; Cell Marque, Merck KGaA, Darmstadt, Germany) and Ki-67 (1:100, clone SP6, Hisztopatológia Kft., Pécs, Hungary), respectively. Heat-mediated antigen retrieval was carried out at pH9. The Ki-67 signal in nuclei was evaluated in PAS-counterstained tissue sections at ×20 objective lens magnification. Twenty-five consecutive glomeruli were read. The signal could have been mesangial, intracapillary, undetermined (either mesangial or intracapillary), endothelial, or podocytic. The glomerular proliferation index was calculated by dividing the total number of Ki67^+^ cells by the number of glomeruli sampled [39]. The mean glomerular diameter was estimated by measuring the maximum glomerular diameter in at least eight profiles exhibiting the vascular pole in the section plane. The parameter value obtained was compared to that measured in three 4-year-old patients with the renal biopsy diagnosis of minimal change nephropathy (age-matched controls). The immunostaining of FN on FFPE sections was performed in the immunohistochemical laboratory of J. U. Becker with polyclonal anti-fibronectin antibody (A 0245, DAKO, Hamburg, Germany) at a dilution of 1:15,000 after enzyme mediated antigen retrieval (AR9551 Bond Wetzlar, Germany).

### 2.3. Renal Biopsy Findings

**Figure 1:**
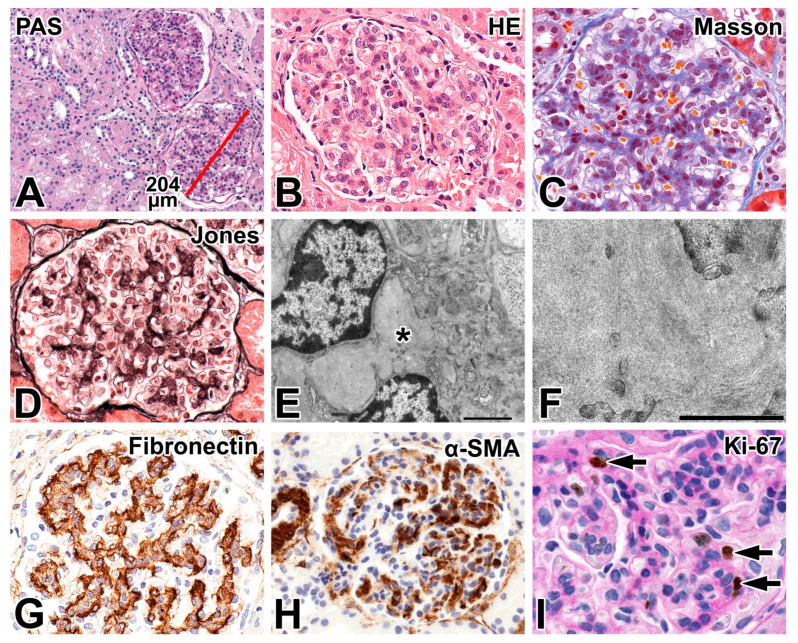
Renal biopsy findings in early childhood-onset fibronectin glomerulopathy. (**A**) Light microscopy revealed markedly enlarged hypercellular glomeruli; the tubules and interstitium appear normal. The diameter of the lower glomerulus: 204 μm (red line). The glomerular size was in the range of that in non-obese adult patients. Periodic acid-Schiff (PAS) reaction. The mesangium was widened by proliferated cells and an expanded matrix area, shown in (**B**–**D**,**G**,**I**). (**B**) The mesangial areas were slightly eosinophilic in hematoxylin and eosin, (**C**) blue in Masson’s trichrome, (**D**) positive in Jones’ methenamine silver, and (**I**) weakly positive in PAS. (**E**) The mesangial area at low magnification with electron microscopy. The expanded matrix is finely granular, and it is surrounded by nuclei and cytoplasmic cell processes of mesangial cells. The area around the asterisk is shown at higher magnification in (**F**). (**F**) Vague fibrils and ill-defined denser areas alternate with lighter areas. (**G**) The strong fibronectin positivity of mesangial areas. (**H**) The strong positivity of mesangial cells with smooth muscle-specific actin (SMA). (**I**) Ki-67 positivity in nuclei of mesangial cells (arrows). The reaction was counterstained with PAS. Original magnification: ×200 for (**A**), 630× for (**B**–**D**,**G**), 5000× for (**E**), 22,000× for (**F**), 400× for (**H**), and 1000× for (**I**).

Patent glomeruli were observed, which were markedly enlarged, and their size was comparable to that encountered in non-obese adults (Figure 1A). The mesangium was diffusely widened by an increase in mesangial cells (2 to 6 nuclei/area) and a mild expansion of the mesangial matrix-area (Figure 1B). The mesangial areas were slightly eosinophilic (Figure 1B), blue in Masson’s trichrome staining (Figure 1C), positive in methenamine silver (Figure 1D), and weakly to moderately positive with the PAS-reaction (Figure 1I). Furthermore, 5–8 leukocytes, predominantly neutrophils, were observed in the glomerular capillary tufts. The tubules, interstitium, and vessels were normal. The Oil Red O and the Congo red stains were negative. The IF evaluation did not reveal glomerular immunoglobulin or complement deposits. Two glomeruli were investigated electron microscopically. The foot processes were diffusely effaced (90%); the filtration slit membranes could be recognized. The microvillous transformation of podocyte cell bodies or dense inclusions in their cytoplasm were not encountered. The glomerular basement membrane (GBM) appeared normal, and the endothelial layer was fenestrated. The mesangial areas were widened by mesangial cell processes and nuclei (Figure 1E). At times, only an expanded matrix was seen, which was finely granular, and its density was not homogenously uniform. At high magnification, the matrix focally exhibited a fibrillary background (Figure 1F). Collagen fibers in small numbers here and there accompanied the mesangial changes. The clinicopathologic features were not obvious for any specific disease entity, and genetically induced glomerular disease was suspected. The FN immunostaining performed in the light of an *FN1* mutation revealed massive positivity in the mesangial areas (Figure 1G), and early childhood-onset FG was diagnosed.

The histopathological features of early childhood-onset FG were characterized by measurements of glomerular size and the assessment of activation and proliferative activity of mesangial cells. The mean glomerular diameter was 196.6 μm (range 190–217); it was 140 μm (range 132–148) in the age-matched controls. The glomerular expression of α-SMA was strong and diffusely present in the mesangia. The number of Ki-67-positive nuclei sampled in glomeruli was mesangial 19 (43.1%), undetermined 11 (25%), intracapillary 10 (22.7%), endothelial 3 (6.8%), and podocytic 1 (2.2%); the glomerular proliferation index was 1.76. Furthermore, Ki-67-positive nuclei in proximal tubular, distal tubular, and collecting ductal cells were seen occasionally. Ki-67-positive nuclei in interstitial cells were infrequently noted.

### 2.4. Genetic Analyses

Genomic DNA was prepared from blood samples using the MagCore Genomic Whole Blood Kit (RBC Bioscience, New Taipei City, Taiwan). Whole exome sequencing (WES) was performed in the patient; the genomic capture was carried out with NEXTERA (Illumina, San Diego, CA, USA); and the massively parallel sequencing was done using the NextSeq500 Sequencer (Illumina, San Diego, CA, USA) in combination with the NextSeq™ 500 High Output Kit (2 × 150 bp). Raw sequence data analyses, including base calling, de-multiplexing, alignment to the GRCh37 human reference genome, and variant calling, were performed using an in-house GATK joint model pipeline. For variant filtration, all disease-causing variants reported in HGMD^®^, ClinVar, along with all variants with minor allele frequency (MAF) of less than 1% in the ExAc database, were considered. The mean coverage of the target regions was 156.5×, and 96.57% of the target regions had >20× coverage. The fraction of low confidence regions (>10% of the region had coverage below 10×) in the analyzed sample was 1.93 percent. Variants that possibly impair the protein sequence, such as the disruption of conserved splice sites, missense, nonsense, read-throughs, or small insertions/deletions, were prioritized. All the relevant inheritance patterns were considered. The candidate pathogenic mutations and their segregation in the family were validated by PCR amplification and Sanger sequencing.

### 2.5. Results of Genetic Testing

**Figure 2:**
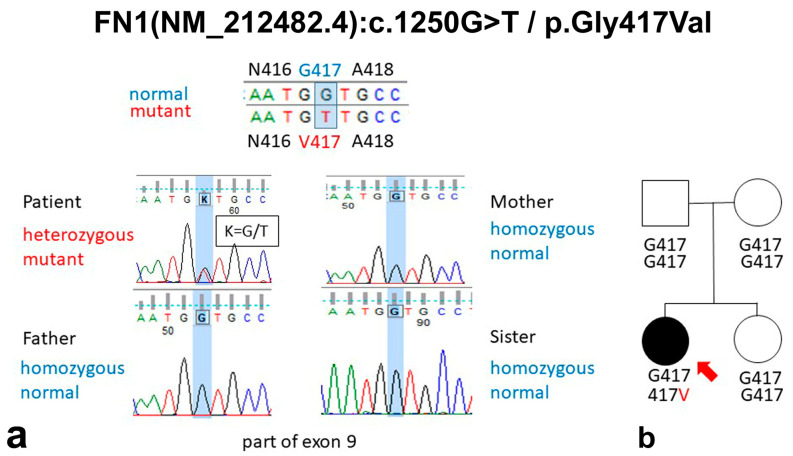
(**a**) A sequenogram of the *FN1* gene exon 9 of the investigated family members. The genetic analysis identified a heterozygous likely pathogenic mutation (NM_212482.4):c.1250G>T/p.Gly417Val) in the *FN1* gene in the index patient. (**b**) Family lineage analyses indicated a de novo mutation since the identified heterozygous FN1 variant was absent in the blood DNA samples of the family members without clinical symptoms. Red arrow indicates the proband.

**Figure 3:**
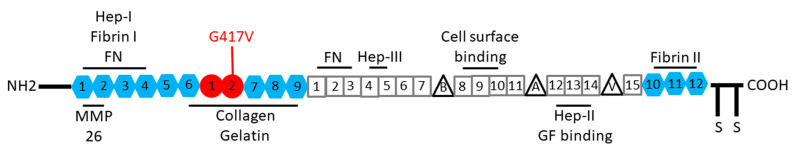
The fibronectin (FN) monomer molecule consists of type I (blue), II (red), and III (white) domains. Extra domains A and B, and variant region (V) are alternative splice sites, differently expressed in both adult and fetal tissues. The binding sites for different proteins are indicated. Pathogenic mutations in fibronectin glomerulopathy affect the heparin-binding domains II/III or only rarely the cell surface binding integrin domain. The single nucleotide change NM_212482.4:c.1250G>T/p.Gly417Val in exon 9 identified in our patient is located in the FN II-2 domain which binds collagen. Abbreviations: MMP 26—metalloprotease 26, GF—growth factor, Hep—heparin, FN—fibronectin.

Next-generation sequencing confirmed by Sanger sequencing identified a single nucleotide change (NM_212482.4:c.1250G>T/p.Gly417Val) in exon 9 in the *FN1* (2q35) gene in a heterozygous state in the patient (Figure 2 and Figure 3). The detected variant is absent in the gnomAD Genome (v4.0), gnomAD Exome (v4.0), Public HGMD, dbSNP(v156), and ClinVar (v20240917) databases, just as in our own national database. To access the conservation level (both amino acid and nucleotide level), the altered sequence was compared with ten other species using MutationTaster2021 [40]. All the investigated mammals (chimpanzee (*Pan troglodytes*), rhesus macaque (*Macaca mulatta*), cat (*Felis catus*), and house mouse (*Mus musculus*) used in this comparison had an identical amino acid composition in this region. Even the red junglefowl (*Gallus gallus*), the Western clawed frog (*Xenopus tropicalis*), and the Japanese pufferfish (*Takifugu rubripes*) have the conserved amino acid Glycin in this position. The zebrafish (*Danio rerio*), the fruit fly (*Drosophila melanogaster*), and the roundworm (*Caenorhabditis elegans*) have no homologous FN1 protein. The in silico predictions were carried out by VarSome [41] and MobiDetails [42]. VarSome and the majority of in-silico prediction algorithms of the MobiDetails tool labeled the variant as likely pathogenic. These include damaging predictions obtained from SIFT, REVEL, ClinPred, Mistic, Meta SVM, and Meta LR; probable damaging predictions obtained from PolyPhen 2 and AlphaMissense; and a tolerated prediction obtained from Fathmm. A segregation analysis of the variant in the family revealed that only the symptomatic index patient had this variant in the *FN1* gene, while asymptomatic relatives did not, suggesting a potential de novo mutation; although, the rare possibility of parental gamete mosaicism cannot be excluded (Figure 2).

### 2.6. A Comparison of the Biopsy Features of Early and Advanced FG

**Figure 4:**
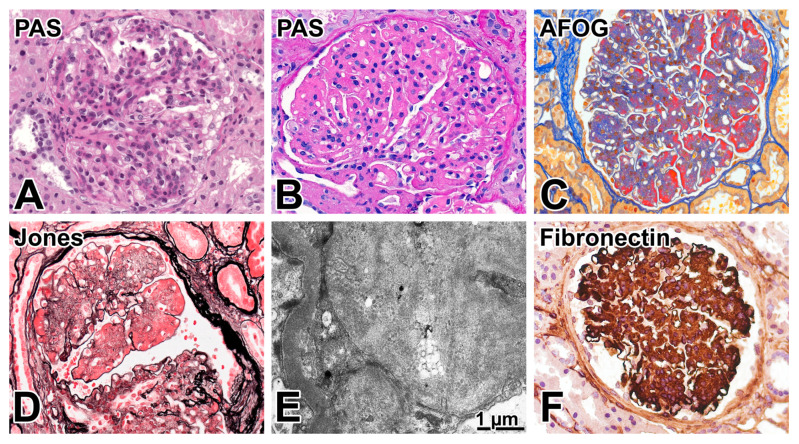
Differences in the histopathological appearance of early vs advanced fibronectin glomerulopathy at-a-glance. (**A**) Mesangial proliferative pattern from early fibronectin glomerulopathy. The mesangial areas did not react intensely with the PAS reaction. PAS, ×200. (**B**–**F**) show the advanced state of fibronectin glomerulopathy diagnosed in an adult patient. (**B**) A lobulated pattern due to a massive mesangial expansion that stained strongly with PAS (×400), (**C**) red with acid fuchsine-orange G stain (×400), and (**D**) negative with Jones’ silver methenamine stain (×400). (**E**) Expanded mesangium due to an abundance of electron-dense granular material, admixed focally with membranous and filamentous structures. Electron microscopy, original magnification, ×8000. (**F**) The strong fibronectin positivity of expanded mesangium, ×400.

We were able to compare the biopsy features of early childhood-onset FG with a case of FG in an adult diagnosed in our institute 16 years earlier. The processing of the biopsy specimen was identical to the pediatric case presented above. The 44-year-old female patient was a Romanian citizen. She was evaluated clinically in Timisoara (Romania) for 0.5 to 2.5 g/d proteinuria, intermittent hematuria, and treated hypertension, which was known about for a year. At the time of renal biopsy performed in Szeged in 2008, her serum creatinine value was 106 μmol/L, and the eGFR value was 55 mL/min/1.73 m^2^. The glomerular deposition of PAS-positive, acid fuchsin orange G-positive, and silver-negative material in a lobular pattern, which was ultrastructurally electron-dense and granular, was highly suggestive of FG. The FN immunostaining performed by our coauthor H. Hopfer concluded the case (Figure 4). The immunostaining protocol in Basel used an anti-human fibronectin antibody (clone IST-4, Sigma Aldrich product no. F0916, Darmstadt, Germany) at a dilution of 1:400 after antigen retrieval with pepsin digestion. The parents of the patient were alive, and they did not have chronic bilateral kidney disease. However, she had a brother who underwent a renal biopsy evaluation in Cluj, Romania at the age of 11 for 1 to 2 g/d proteinuria. Chronic glomerular disease with “membranoproliferative features” was reported. He died unexpectedly at the age of 17. A revision of the histopathological slides of the brother was not possible but her brother could have had FG. The genetic testing of the family for *FN1* mutations was not available in this part of Europe at that time.

## 3. Discussion

In pediatric clinical nephrology practice, there is a tendency to replace the diagnostic kidney biopsy with exome sequencing on suspicion of an inherited kidney disease [43]. Our approach is to conduct genetic testing first if there is a clear family history of renal disease, a clear suspicion of an inherited kidney disease or if extrarenal features are present. We perform a biopsy first if extrarenal features are absent and the family history is negative; we applied this protocol in the case presented. As shown, the biopsy alterations did not match any diagnostic entity and in this way suggested a genetically induced glomerular disease. Nevertheless, the normal GBM, the lack of focal segmental/global glomerulosclerosis along with the normal ultrastructural morphology of podocyte cell bodies, and the normal tubulointerstitium guided our planning of how to construct the genetic testing. This also included the investigation of the *FN1* gene, because a causative *FN1* mutation was identified recently in an international patient cohort of children with steroid-resistant proteinuria and hematuria [44]. In light of the identified likely pathogenetic variant of the *FN1* gene, we performed immunohistochemical staining for FN, and the strong mesangial positivity diagnosed the case as FG with nontypical histopathological features.

The deposited abnormal FN protein should have activated the mesangial cells since strong and diffuse expression of α-SMA and markedly increased proliferation activity were detected in these cells. The calculated glomerular Ki-67 proliferation index of 1.76 was high, comparable to that reported in the setting of “acute glomerulonephritis” by Alpers et al. in 1992 (mean: 1.95; post-infectious 3, cryoglobulinemic, and not otherwise classifiable 1) [39]. The FN deposition, along with the vigorous proliferative response induced a massive enlargement of glomeruli; the size of glomeruli in our 4-year-old patient lay in the range of non-obese adult patients. The changes resulting in glomerulomegaly somehow induced glomerular hyperfiltration, because eGFR values above the threshold of 135 mL/min/1.73 m^2^ [45] characterized all the visits in the follow-up period. Although we did not know the exact pathophysiologic events of glomerular hyperfiltration in our patient, we recall that glomerulomegaly-associated conditions, such as early diabetic nephropathy and obesity, are commonly associated with glomerular hyperfiltration [46].

Two factors may explain why the renal biopsy findings were not typical for FG in our patient. The first factor was the timing of the biopsy evaluation. A deposition disease, such as renal amyloidosis or diabetic nephropathy diagnosed in the early phase or in the advanced phase commonly manifests itself with different clinical and pathologic features. FG in our patient was obviously in the initial phase since it was present with asymptomatic proteinuria discovered accidentally and the renal biopsy evaluation did not raise the suspicion of FN deposition disease. In retrospect, the mild acellular expansion of the mesangial matrix area observed electron microscopically might have indicated early, just developing FG, but the simultaneous presence of mesangial cell proliferation and the non-distinct appearance of mesangial matrix masked the diagnostic relevance of the mesangial expansion itself. We read about two FG patients in the English medical literature (reviewed in the paragraphs below) who also had an early clinical manifestation of the disease and bland renal biopsy changes [16,24]. In contrast, an overwhelming majority of FG patients have the fully blown disease, manifesting itself with nephrotic-range proteinuria or sometimes with a decade-long history of proteinuria and distinct histopathological abnormalities in the renal biopsy specimen. The strong glomerular FN positivity confirms the diagnosis [9,10,16,25,26].

The second factor contributing to the nontypical biopsy features in our patient might be the physicochemical properties of the mutated protein itself. The mutation occurred at a very conserved position involving the collagen-binding site in the FN-II domain, which presumably affected the molecular conformation of FN-protein, FN fibrillogenesis, or FN matrix assembly. Although these features were not investigated in the present communication because they require a specially equipped, basic science laboratory, the individual features of mutated FN protein might influence the nonconventional renal biopsy morphology and manifestation of the disease already at the toddler stage. Significant proteinuria, intermittent microhematuria, hyperfiltrating glomeruli, and stable renal function characterized her kidney disease in the follow-up period of almost two years.

A literature search on FG found an English abstract of a Japanese publication from 1991 on five cases from four families with FG [47]. The authors performed a morphometrical analysis of FG taken from a 4-year-old, a 19-year-old, a 27-year-old, a 58-year-old, and a 75-year-old patient. All of them had a much higher value (201 times) of mean glomerular-tuft area and a higher mean number (2.0 times) of mesangial cells. The younger patients exhibited a markedly higher value of the mean glomerular tuft area and mean number of mesangial cells than the older patients. The authors concluded that the enlargement of glomeruli was caused by the intraglomerular accumulation of FN, collagen IV, and laminin and proliferated mesangial cells. The conspicuous mesangial cell proliferation observed in the younger Japanese patients and in our 4-year-old patient suggests that the deposition of abnormal FN can trigger a marked proliferative response in mesangial cells that ceases when the lobulation of glomeruli starts.

Now, we shall review the published cases of early childhood-onset FG. In the report of Yang and Shen [24], a Chinese boy, aged 2 years and 2 months was evaluated for hematuria and occasional proteinuria. The renal symptoms lasted 22 months. The renal biopsy sample did not reveal any pathologic changes light microscopically. With EM, the GBM was of uneven thickness. The “stratum compactum” of the basement membrane was thickened, with a small part having a tear-like and cobweb-like morphology. Alport nephropathy was considered initially. However, the genetic test excluded it and disclosed a heterozygous mutation of the *FN1* gene located in the FN III-5 domain. Immunostaining for FN was not carried out retrospectively, and therefore we cannot say whether the mesangia had accumulated FN or not. The follow-up at 3 years and 4 months revealed persistent microhematuria and intermittent proteinuria, with no abnormality in renal function. The child’s mother had a history of hematuria and nephrolithiasis for 5 years and she displayed the same heterozygous mutation of the *FN1* gene. We interpreted the case in retrospect as early childhood-onset FG without typical histopathological changes.

In the paper by Niimi et al. [38], a 3-year-old Japanese boy was evaluated for nephrotic-range proteinuria, microhematuria, and an extremely high blood pressure value. The patient had pretibial and facial edema. The duration of the symptoms was not reported. Headaches presumably related to hypertension lasted for at least a month. Blood urea nitrogen and creatinine concentrations were within normal limits. Upon nifedipine and captopril treatment, the hypertension and edema improved; and the proteinuria decreased to 0.5 g/day. A family history of renal disease was positive in 12 members of three generations. However, none of them had undergone a renal biopsy. The renal biopsy evaluation of the boy revealed enlarged glomeruli with expanded mesangium. The mesangial cells were not increased in number. Capillaries were peripherally displaced by the expansion of the mesangial area, and the basement membrane was moderately and irregularly thickened. The glomeruli were negative for immune deposits. EM showed electron-dense, fine granular deposits in the mesangial stalk and in the subendothelial space of capillary loops. Immunohistochemistry revealed prominent FN positivity at the sites of the deposits and FG was diagnosed. Curiously, progressive renal failure was not noted within the follow-up period of 7 years. We classified the case in the histological subset of a lobular pattern in retrospect.

In the paper by Klair et al., a 36-year-old male patient was described who had progressive chronic kidney disease after a kidney transplant [16]. During a workup of developmental delay at age 5, proteinuria was found incidentally and the subsequent kidney biopsy evaluation diagnosed type 1 MPGN even though he had normal complement levels. At age 23 he received a living unrelated donor kidney transplant. Two months after transplantation, his serum creatinine level was progressively increased to 168 μmol/L, and proteinuria of 0.6 g/day was detected. The renal transplant biopsy evaluation did not reveal rejection or glomerular disease, and in particular, there were no deposits by IF and EM. After a stable period of allograft function, the proteinuria increased to 1.7 g/day, and the serum creatinine value increased to 194 μmol/L at 12 years post-transplant. A second biopsy was performed. This time an MPGN pattern of injury with lobular accentuation was observed with marked mesangial expansion by PAS-positive and silver-negative material. Glomerular immune deposits were not detected. EM revealed massive electron-dense deposits in mesangial and subendothelial regions with a focal, vague fibrillar appearance. The changes suggested FG and prompted immunostaining of FN, which was strongly positive in the deposits. Genetic testing found a previously identified pathogenic alteration [6], and the diagnosis of MPGN type 1 was corrected to FG. His mother died in her 30 s and she was diagnosed with MPGN on autopsy. It is likely that she had FG as well. This unique case report also stated that at the very beginning of recurrent FG (proteinuria was 0.6 g/d), the renal transplant biopsy features were unremarkable.

As reviewed here, the renal symptoms were diverse in early childhood-onset FG: asymptomatic proteinuria in our patient and in the 5-year-old boy mentioned above, hematuria and intermittent proteinuria in the Chinese boy, and nephrotic syndrome in the Japanese boy. The histological pattern of FG, lobular, MPGN-like, mesangial proliferative, or no change, also differed in these cases. The varied clinical and histological manifestations of FG highlight the importance of characteristics of mutated FN1-protein, shown in a recent genotypic-phenotypic correlation study [20]. In the biopsy sample of a 13-year-old Chinese boy evaluated for recurrent proteinuria and microhematuria, there were no typical large mesangial and subendothelial FN deposits, mesangial cell proliferation, inflammatory cell infiltration, or global glomerular or segmental sclerosis. EM revealed thinned GBM-s and FN immunohistochemistry demonstrated increased FN staining along the glomerular capillaries. The morphologically thin basement membrane nephropathy was associated with a novel gain-of-function mutation in the *FN1* gene (c.3415G>A) inherited in an autosomal dominant fashion in the family. This variant FN induced decreased FN binding to collagen IV alfa 3 and 4 and increased the beta-1 integrin expression of glomeruli.

## 4. Conclusions

The renal biopsy evaluation of a 4-year-old girl with asymptomatic proteinuria, absence of extrarenal features, and negative family history could not explain the cause of proteinuria. Glomerulomegaly and the proliferation of intrinsic glomerular cells, especially mesangial cells were the main histologic abnormalities. The genetic testing identified a de novo single nucleotide change (Gly417Val) in the *FN1* gene in heterozygous form, viewed as a likely pathogenic mutation. The immunohistochemical staining for FN revealed strong mesangial positivity and hence FG was diagnosed. Nephrotic range proteinuria, intermittent microhematuria, glomerular hyperfiltration, and preserved renal function characterized the follow-up period. The case was exceptional because the likely pathogenic mutation of the *FN1* gene was located at the collagen-binding site of the FN II-2 domain, not previously identified; the disease manifested itself at an unusually young age; and the mesangial proliferative pattern observed in the biopsy specimen expanded the histological spectrum of FG. Hence nephrologists in the future should also consider FG among the monogenic causes of proteinuric kidney diseases in children [44]. Further studies are needed to better understand the pathogenesis of FN1-protein deposition in glomeruli and the mutational profile of the clinical disease.

## Figures and Tables

**Table 1:** Laboratory results of the kidney disease. The eGFR values above 135 mL/min/1.73 m^2^ indicate glomerular hyperfiltration.

	First Admission	5 Weeks Later (Renal Biopsy)	6 Months Later	12 Months Later	15 Months Later	29 Months Later
Serum albumin (g/L)	37	36	30	24	28	29
Serum total protein (g/L)	66	62	57	53	57	59
Serum creatinine (μmol/L)	20	18	16	15	15	15
Serum carbamide (mmol/L)	3.2	3.3	2.8	3.4	2.7	2.3
Serum parathormone (pmol/L)	-	-	1.3	3.1	-	-
Serum cholesterol (mmol/L)	10.44	9.36	-	5.11	-	7.88
Serum triglyceride mmol/L)	1.8	1.03	-	12.65	-	7.48
Urine total protein (mg/dL)	19.3	150.4	174.6	375.4	183.6	135.5
Urine creatinine (mmol/L)	1086	4499	3521	5143	6382	4137
Urine total protein-to-creatinine ratio (mg/mmol)	177.72	334.30	317.81	729.92	287.68	327.53
Estimated GFR (bedside Schwartz formula, mL/min/1.73 m^2^)	>135	>135	>135	>135	>135	>135
Urine ß-2-microglobulin (μg/L)	23.00	-	48.00	-	-	-
Microhematuria	no	no	no	yes	yes	no

## Data Availability

Sequence data sets are unavailable due to privacy and ethical restrictions.
